# Does Frailty Predict Cognitive and Functional Deficits After Nine Years?

**DOI:** 10.1002/gps.70104

**Published:** 2025-05-30

**Authors:** Beatriz Raz Franco de Santana, Daniela de Assumpção, Flávia Silva Arbex Borim, Ivan Aprahamian, Ligiana Pires Corona, Samila Sathler Tavares Batistoni, Deusivania Vieira da Silva Falcão, Meire Cachioni, Ruth Caldeira de Melo, Anita Liberalesso Neri, Monica Sanches Yassuda

**Affiliations:** ^1^ Department of Gerontology Faculty of Medical Sciences Post Graduation Program in Gerontology Universidade Estadual de Campinas Campinas Brazil; ^2^ Department of Gerontology Faculty of Medical Sciences Faculdade de Medicina de Jundiaí Post Graduation Program in Gerontology Jundiai Brazil; ^3^ Department of Gerontology School of Communications and Arts Post Graduation Program in Gerontology Universidade de São Paulo São Paulo Brazil

**Keywords:** aging, cognition, dementia, frailty, longitudinal studies

## Abstract

**Objectives:**

To identify the variables at baseline, including physical frailty, that might predict cognitive and functional deficits in a 9‐year follow‐up.

**Methods:**

This investigation included participants from the FIBRA study in Campinas city and Ermelino Matarazzo, subdistrict of São Paulo city, with complete data collected at baseline and follow‐up for the variables sex, age, education, frailty phenotype, number of chronic diseases, and tobacco and alcohol use. Of the initial 1284 participants at baseline, 98 that exhibited cognitive impairment were excluded. At follow‐up, 451 participants were located and reinterviewed and 85 scored below the cut‐off on the Mini‐Mental State Exam (MMSE), of which 45 also presented functional deficit.

**Results:**

The follow‐up subsample comprised predominantly participants that were female (68.1%), aged 65–74 years (71.6%), and had low education (0–4 years of education, 75.6%). At baseline, 35.5% were non‐frail, 57.0% pre‐frail and 7.5% frail, whereas at follow‐up, 29.4% were non‐frail, 62.3% pre‐frail and 8.3% frail. Logistic regression showed that age and education but not frailty at baseline were associated with cognitive and functional deficits at follow‐up.

**Conclusions:**

Higher age and lower education at baseline were predictors of cognitive and functional deficits after 9 years, whereas frailty was not. Further longitudinal studies should be conducted to elucidate the factors predicting cognitive and functional decline in low‐and middle‐income countries.

**Summary**

- Frailty at baseline was not a significant predictor of cognitive and functional impairment over 9 years.
- Higher age and lower educational level at baseline were associated with increased risk of cognitive and functional decline.
- The findings contrast with previous international studies that reported frailty as a predictor of dementia, suggesting possible population or methodological differences.
- Further longitudinal research is needed in low‐ and middle‐income countries to clarify predictors of cognitive and functional decline and guide preventive strategies.

## 1 Introduction

The rising prevalence of dementia is one of the more evident effects of an aging world population. The disease represents one of the greatest global health challenges in the 21^st^ century [1]. According to data from Alzheimer's Disease International, in 2019 [2], there were around 55 million people living with dementia, mostly from low‐and middle‐income countries [3]. Globally, an estimated 78 million people will be affected by dementia by 2030, with this figure rising to 139 million by 2050 [3]. Currently, it is estimated that 1.8 million people live with dementia in Brazil [4].

A systematic review with data from Latin America and the Caribbean showed that the prevalence of dementia in the region was higher than in high‐income countries [5]. There was higher prevalence among women, low‐educated individuals, those living in rural areas, and who were older [6]. Older adults living in Brazil are affected by similar risk factors for dementia to those identified in countries in Latin America and the Caribbean [7, 8, 9].

Sociodemographic characteristics, genetic factors, and chronic non‐communicable diseases, such as diabetes, hypertension and hypercholesterolemia, contribute significantly to cognitive decline [10, 11, 12]. Psychological factors such as depression, neuroticism and purpose in life [13, 14] have also been associated with cognitive deficits [15]. In Brazil, Suemoto et al. identified the main risk factors for dementia as low education (7.7%), hypertension (7.6%) and hearing loss (6.8%) [16].

Physical frailty, defined as lower capacity to cope with stressors [15, 16] was identified as a risk factor for cognitive impairment and dementia [17, 18]. A study on frailty of around 197,000 older adults from the UK Biobank investigated the relationships between frailty, lifestyle, dementia risk and dementia incidence [19]. During an 8‐year follow‐up, 3.5% of participants developed dementia, and frailty was associated with increased dementia risk independently of genetic risk and mediated 44% of the relationship between healthy lifestyle behaviors and dementia risk. Also, participants with elevated genetic risk and high frailty had a 5.8 times greater risk of incident dementia compared to those with low genetic risk and low frailty [19].

The Frailty in Brazilian Older Adults (FIBRA) study investigated the association of frailty with aspects of physical and psychological health, including cognition, in individuals aged ≥ 65 years [20, 21, 22]. After a 9‐year follow‐up, there was an increase in the number of participants with cognitive deficit (17.7% vs. 23.5%) and frailty (9.8% vs. 24.5%) [20, 21]. Longitudinal studies investigating the impact of frailty on cognitive functioning are not available in Brazil. Therefore, the objective of the present study was to investigate whether physical frailty at baseline increases the risk of a decline in global cognitive scores and in activities of daily living (ADL) after 9 years. In the present analysis, the hypothesis tested was that, in the presence of sociodemographic and health variables, frailty at baseline would be associated with cognitive and functional deficits after 9 years.

## 2 Methods

The FIBRA [20] is a population‐based study whose goal was to investigate associations of frailty with sociodemographic and mental and physical health variables in Brazilian older adults aged ≥ 65 years. In the baseline evaluation (2008–2009), older adults recruited in randomized census sectors, from seven Brazilian cites, were invited to take part in data collection sessions (mean duration 90 min) at community centers. Eligibility criteria were being aged ≥ 65 years, a permanent resident of the city and living at home. Individuals exhibiting severe stroke complications, severe or unstable Parkinson's disease, severe visual or hearing deficits, who were bedridden, terminal, or with cancer undergoing chemotherapy treatment, were excluded. For further details on these and other aspects of the FIBRA study see Neri, Yassuda et al. [20]. The dataset of the FIBRA study is available at https://doi.org/10.25824/redu/TTCQJJ.

In 2016 and 2017, a 9‐year follow‐up was carried out at two sites involved in the initial study [21], applying the same eligibility and exclusion criteria used at baseline. For participants who scored below the cut‐off on the Mini‐Mental State Examination (MMSE), the interview was conducted with a family member or close informant.

The study included participants of the Campinas and Ermelino Matarazzo (São Paulo) surveys with complete data at baseline and follow‐up for age, sex, education, MMSE, functional status and frailty. Of the total participants (*n* = 1284) at baseline for the two locations, 549 (42.7% of original sample) were located and reinterviewed at follow‐up. Overall, 192 (14.9%) individuals had died, while 543 (42.4%) were lost to follow‐up due to refusal to participate, not found at former address, or for other reasons (Neri et al., 2022). Several participants (*n* = 314, 57.9%) were not located at the addresses and phone numbers indicated at baseline. Such addresses were visited three times at different time periods, and neighbors and nearby stores were visited in an attempt to locate participants or have information about their new address or phone number.

**FIGURE 1:**
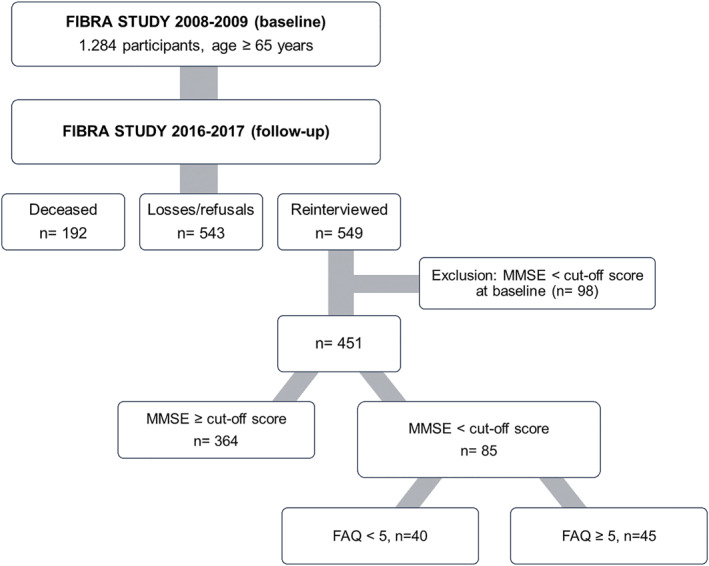
Flow diagram of sample composition. FIBRA study, Campinas and Ermelino Matarazzo, São Paulo state, Brazil, 2008/2009 and 2016/2017. FAQ = Functional Activities Questionnaire, MMSE = Mini Mental State Examination.

Of the 549 participants with data at follow‐up, 98 were excluded for presenting cognitive impairment on the MMSE at baseline (*n* = 451). Cognitive deficit at follow‐up was assessed with a MMSE score below the cut‐off (*n* = 87). There was information missing from two participants. Among those (*n* = 85), 45 participants had Functional Activity Questionnaire (FAQ) score ≥ 5. The MMSE was completed by the participants and the FAQ was completed by a family member or close informant. The flow diagram in Figure 1 shows the composition of the present study sample.

### 2.1 Variables and Measures

The MMSE [22] is an instrument for assessing global cognition that yields a total score of 0–30 points. Cognitive decline was defined using the following cut‐off points: Illiterate = 17 points; 1–4 years of education = 22 points; 4–8 years = 24 points; and ≥ 9 years = 26 points. These cut‐off scores represent means for the education ranges reported by Brucki et al. [23], minus 1 standard deviation.

The FAQ [24] measures performance for 11 instrumental activities of daily living (IADLs), such as managing personal finances and preparing meals. There are 4 response options: 0—independent/normal; 1—some difficulty; 2—requires assistance; 3—cannot do/dependent. Scores range from 0 (minimum) to 30 (maximum). Lower overall score indicates higher level of independence. In the present analysis, a FAQ score of ≥ 5 points was considered indicative of functional limitation [24].

The following sociodemographic variables at baseline were extracted from the FIBRA study database: sex (male, female), age group (65–69, 70–74 and ≥ 75 years) and education (no formal schooling, one to four and ≥ 5 years of education).

Health‐related behaviors and chronic diseases extracted from the database were: tobacco use (never smoked, current smoker, former smoker) and alcohol use (non‐drinker, drinks 1–4 times a month, ≥ 2 times a week). For chronic diseases, participants were asked whether they had been clinically diagnosed in the last 12 months with the following diseases: arterial hypertension, arthritis/rheumatism, diabetes mellitus, osteoporosis, heart disease, depression, lung diseases and cancer. The number of chronic diseases was categorized into 0–1, 2–3, or > 3.

Frailty was assessed by the phenotype of Fried, Tangen et al. [25] with five components: (1) Unintentional weight loss in past 12 months on interview, corresponding to 4.5 kg or 5% of body weight; (2) Fatigue was indicated by the answer always or almost always on two items on fatigue derived from the Center for Epidemiologic Studies Depression Scale (CES‐D); (3) Low hand‐grip strength, expressed as kg/force, below the first quintile of the distribution of means for the sample, measured by three consecutive attempts pulling the handle of a Jamar dynamometer, with values adjusted for sex and body mass index (BMI); (4) Slow gait indicated by mean time (in seconds) to walk 4.6 m in a straight line using usual stride, with value above the 80^th^ percentile of the distribution for the sample, adjusting for sex and height; and (5) Low physical activity level defined as weekly energy expenditure below the first quintile of the distribution of metabolic units expended by the participants in cumulative weekly domestic activities and physical exercises at low, moderate and vigorous intensity, as per response options for items selected from the Minnesota Leisure Time Activities Questionnaire. Individuals whose metabolic equivalent (METs) values were below the first quintile of the sample, corrected for sex, were rated as having a low level of physical activity. The procedures, criteria, cut‐off scores and adjusting variables adopted were those described by Fried, Tangen et al. [25]. Participants that scored on one or 2 criteria were classified as pre‐frail, ≥ 3 criteria as frail, and none of the criteria as non‐frail.

The 2008–2009 FIBRA baseline study was approved by the Research Ethics Committee of the Universidade Estadual de Campinas (UNICAMP), under permits 208/2007 and 907.575. The follow‐up study was approved under permit numbers 1.332.651 and 2.952.507 at UNICAMP. Additionally, the study was approved by the local Research Ethics Committees. All respondents signed the Free and Informed Consent Form.

### 2.2 Data Analysis

The sample was characterized by descriptive analysis (absolute frequency and relative percent) of variables at baseline. Associations of cognitive and functional impairment with the independent variables were explored using Pearson's chi‐square or Fisher's Exact tests, with a significance level of 5%.

On the logistic regression, the outcome investigated was having cognitive and functional impairment at follow‐up, while exposure variables were frailty status adjusted for sex, age and education. Further health and lifestyle variables were not entered into the model due to the limitation in sample size at follow‐up.

In the multiple regression analysis, the regression coefficient values and their respective 95% confidence intervals were presented, along with the *p*‐values from the Wald test and Nagelkerke's *R* ^2^ (ranging from 0 to 1, where values closer to one indicate a better explanatory power of the model). The model's significance was assessed using the Hosmer and Lemeshow test (a *p*‐value > 0.05 suggests a good fit), as well as information from the classification table.

The analyses were conducted using the statistical software Stata, version 15 (https://www.stata.com).

## 3 Results

Data for 451 participants aged ≥ 65 years with MMSE score above cut off score at baseline (2008–2009) were analyzed. With regard to sociodemographic and clinical characteristics at baseline, the sample comprised predominantly individuals who were female (68.1%), aged 65–74 years (71.6%), had formal schooling or 1–4 years of education (75.6%), never smoked (57.7%), were non‐users of alcoholic beverages (33.0%) and had ≥ 2 chronic diseases (69.1%). With regard to frailty at baseline, 35.5% were classified as non‐frail, 57.0% as pre‐frail, and 7.5% as frail (Table 1).

**TABLE 1:** Sociodemographic and clinical characteristics of sample at baseline (*n* = 451) and participants with cognitive (*n* = 40) and cognitive and functional deficits at follow‐up (*n* = 45). FIBRA Study, Campinas and Ermelino Matarazzo, São Paulo state, Brazil, 2008–2009 and 2016–2017.

Variables	*n* = 451 (%)	Cognitive deficit	Cognitive and functional deficit
		*n* = 40 (%)	*n* = 45 (%)
Sex		*p* = 0.314^a^	
Male	144 (31.9)	11 (27.5)	17 (37.8)
Female	307 (68.1)	29 (72.5)	28 (62.2)
Age (years)		*p* = 0.028^a^	
65–69	169 (37.5)	20 (50.0)	10 (22.2)
70–74	154 (34.1)	8 (20.0)	14 (31.1)
≥ 75	128 (28.4)	12 (30.0)	21 (46.7)
Education (years)		*p* = 0.167^a^	
Illiterate	74 (16.4)	5 (12.5)	13 (28.9)
1–4	267 (59.2)	27 (67.5)	26 (57.8)
≥ 5	110 (24.4)	8 (20.0)	6 (13.3)
Frailty		*p* = 0.302^b^	
Non‐frail	160 (35.5)	15 (37.5)	10 (22.2)
Pre‐frail	257 (57.0)	22 (55.0)	31 (68.9)
Frail	34 (7.5)	3 (7.5)	4 (8.9)
Number of chronic diseases		*p* = 0.393^a^	
0–1	138 (30.9)	13 (32.5)	10 (22.7)
2	132 (29.6)	8 (20.0)	14 (31.8)
≥ 3	176 (39.5)	19 (47.5)	20 (45.5)
Tobacco use		*p* = 0.544^b^	
Never smoked	258 (57.7)	23 (57.5)	20 (45.4)
Current smoker	42 (9.4)	4 (10.0)	5 (11.4)
Former smoker	147 (32.9)	13 (32.5)	19 (43.2)
Alcohol use		*p* = 0.209^a^	
Drinker	296 (67.0)	14 (35.0)	10 (23.3)
Non‐drinker	146 (33.0)	25 (62.5)	33 (76.7)

^a^ *p‐*value from Pearson's chi‐square test, bolded *p* < 0.05.

^b^ *p*‐value from Fisher's exact test.

Between baseline and follow‐up, a total of 87 participants exhibited cognitive impairment (19.3%), of which 45 (52.9%) showed both cognitive and functional deficits. Participants with scores suggesting impairment on the MMSE at 9‐year follow‐up, stratified into those with cognitive (*n* = 40) and cognitive and functional deficits (*n* = 45), are presented in Table 1. The incidence of cognitive and functional deficits was proportionally greater in individuals aged ≥ 70 years (63.6%) compared to those aged < 70 years (33.3%).

The results of logistic regression, with outcome variable of possible cognitive and functional impairment and exposure variable of frailty status, adjusted for sex, age and education, revealed that frailty at baseline was not associated with possible cognitive and functional impairment at follow‐up (Table 2). The variable age and education showed a significant association with cognitive and functional impairment at follow‐up, with greater risk among participants who were older and less educated at baseline.

**TABLE 2:** Logistic regression for cognitive and functional impairment according to sociodemographic variables and frailty. FIBRA Study, Campinas and Ermelino Matarazzo, São Paulo state, Brazil, 2008‐2009 and 2016‐2017.

Variables	Odds Ratio	95% CI	*p*‐value
Frailty (ref. *Non‐frail)*			
Pre‐frail	2.30	0.77; 6.90	0.136
Frail	1.31	0.18; 9.31	0.785
Sex (ref. Male)			
Female	0.57	0.19; 1.70	0.315
Age group (ref. 65–69 years)			
70–74 years	3.80	1.12; 12,91	**0.032**
≥ 75 years	3.81	1.22; 11.89	**0.021**
Education (ref. no formal schooling)			
1–4 years	0.29	0.08; 1.10	0.069
≥ 5 years	0.22	0.05; 0,99	**0.049**

Classification	Predictive		
	Cognitive and functional impairment		Total
	Yes	No	
Yes	31	14	45
No	14	26	40
Total	45	40	85

*Note:* Ref.: Reference category used for comparison. 95% CI: 95% Confidence Interval; *p*‐value from Wald test, bolded *p* < 0.05. Nagelkerke's *R* ^2^ = 0.35. *p* (teste de Hosmer‐Lemeshow) = 0.936.

*Note:* Classification table: sensitivity = 68.89%, specificity = 65.00%, positive predictive value = 68.89%, negative predictive value = 65.00%.

## 4 Discussion

The objective of the present study was to assess whether physical frailty at baseline was associated with cognitive and functional impairment after 9 years. The analysis failed to confirm this association, where greater age and lower education at baseline were found to be associated with cognitive and functional impairment at follow‐up. In contrast with the present results, previous studies have found a significant association between frailty and risk for dementia [26, 27]. e.g., in the English Longitudinal Study of Aging [26], a total of 8722 older adults were followed up every 2 years for a total of 14 years. After adjusting for sex and age, participants who were pre‐frail or frail had a significantly greater risk of developing dementia than non‐frail individuals. After adjusting for socioeconomic level, education and living alone, the associations between frailty and dementia were weaker, but still statistically significant [27]. In a 5‐year longitudinal study of 2022 Chinese older adults, frailty status was found to be a more robust predictor of dementia compared to other variables [28].

In the systematic review of Borges et al. [29], the relationship between frailty and cognitive disorders was explored, and included longitudinal studies. The review selected six studies from Europe, Asia and North America, involving a total study population of 14,657 participants with a mean follow‐up of 5.33 years. Significant associations between frailty and the incidence of cognitive impairments were observed. The authors concluded that frail older adults were at greater risk of developing cognitive disorders, especially vascular dementia, compared with their non‐frail counterparts [29]^.^.

However, other variables may play a role in the association between frailty and cognitive outcomes. For instance, Gray et al. [30] found a significant association between frailty and dementia risk when adjusted for demographic variables (e.g., age, sex), yet, the association did not remain significant in a fully adjusted model, which included other risk factors (such as cardiovascular health and previous cognitive function). Furthermore, it was observed that the association between frailty and dementia remained only for non‐Alzheimer's dementia. Results from Rogers and Pascual‐Leone [31] suggested that frailty may not be useful as a predictor for the progression from mild cognitive impairment (MCI) to dementia as the vulnerabilities associated with frailty may reflect underlying brain diseases, rather than being an independent predictor of dementia. Finally, N. T. Rogers et al. [26] showed a higher risk for dementia among frail and pre‐frail participants, however, this association was only significant for individuals with higher cognitive function at baseline (upper three quartiles of global cognitive function). This finding suggests that frailty may not be a significant predictor of dementia for those with low cognitive performance.

In the present analysis, greater age at baseline was associated with higher risk for cognitive and functional impairment. The association between greater age and dementia risk is widely acknowledged and has been documented in many previous studies. For example, a longitudinal study [28] of 531 older adults, 65 of whom had mild cognitive impairment (MCI) on the initial assessment, found a conversion rate of 32% to dementia in the subsample with MCI. The factors predicting conversion to dementia were greater age and lower educational level. Other factors including smoking, alcohol use and clinical comorbidities, such as diabetes and vascular diseases, were also analyzed, but did not predict cognitive decline over time [28]. The cohort study of Li et al. [27] of Chinese older adults aged ≥ 65 years showed that the 5‐year cumulative incidence rate of dementia was higher in frail than non‐frail individuals, irrespective of the definition of frailty used [27]. This association was especially significant among participants in the third quartile of the age distribution.

In the present analysis, education was also a significant predictive factor of cognitive and functional impairment over the 9‐year follow‐up period. Longitudinal follow‐up studies in Brazil and other regions have shown an association of lower educational level with higher incidence of dementia [8, 32, 33]. In a cross‐sectional study, Suemoto, Bertola et al. [34] investigated the association of education, type of occupation and cognitive impairment of 1023 Brazilians, 77% of whom had less than 5 years of education and 56% held non‐technical jobs. Greater educational level was associated with higher cognitive performance, whereas type of occupation showed no significant association with cognition, even after adjusting for factors such as age, sex and neuropathologic injuries [34].

It is also important to highlight that education is considered a proxy measure of cognitive reserve which might delay the onset of cognitive and functional decline, even in the presence of neurological diseases [35]. Cognitive reserve is a theoretical construct which describes the brain's capacity to cope with aging and age‐related diseases more effectively [36]. A recent systematic review showed that higher education is a consistent protective factor against cognitive impairment [37].

This study has some limitations. For instance, the small subsample in the follow‐up assessment after 9 years may have been less representative of the original sample, as is the case in most longitudinal studies with long follow‐up periods [38]. The lack of interim assessments during the follow‐up period also constitutes a limitation. Another limitation of the present study is the absence of a clinical diagnosis of dementia, which was instead estimated based on cognitive and functional deficits assessed with questionnaires. We acknowledge that the lack of a clinical diagnosis may hypothetically explain the negative findings for the predictive value of frailty for cognitive and functional impairment. In addition, we recognize that the use of the MMSE may have prevented the identification of participants with MCI and the study of individual cognitive domains. We further acknowledge that the FAQ is an indirect measure of ADL and it may be biased by the personal perceptions of the respondent. Future studies can overcome these limitations by including follow‐up evaluations at shorter intervals and by clinically diagnosing dementia based on more comprehensive cognitive tests, laboratory exams and neuroimaging. On the other hand, as strengths of the study we highlight its longitudinal design, the fact that the sample size was favorable for the performed analyses and, most importantly, that follow‐up time was adequate for examining any new cases with cognitive impairments.

## 5 Conclusions

In the present study, frailty did not predict cognitive and functional impairment at 9‐year follow‐up. However, older age and lower education increased the odds of developing frailty. Further longitudinal studies should be conducted to elucidate the factors predicting cognitive and functional decline in low‐to‐middle income countries, thereby contributing to the development of future actions for intervention and prevention of these declines in the older population.

## Conflicts of Interest

The authors declare no conflicts of interest.

## Data Availability

The data that support the findings of this study are openly available in REDU UNICAMP at https://doi.org/10.25824/redu/TTCQJJ.
